# Time-Dependent Controlled Release of Ferulic Acid from Surface-Modified Hollow Nanoporous Silica Particles

**DOI:** 10.3390/ijms241310560

**Published:** 2023-06-23

**Authors:** Tetsuo Yamaguchi, Taeho Kim, Jin-Kuen Park, Jae-Min Oh

**Affiliations:** 1 Department of Energy and Materials Engineering, Dongguk University, Seoul 04620, Republic of Korea; tetsuo.y@dgu.ac.kr (T.Y.); taeho0408@naver.com (T.K.); 2 Department of Chemistry, Hankuk University of Foreign Studies, Yongin 17035, Republic of Korea

**Keywords:** mesoporous silica, drug delivery, hollow silica particle, surface functionalization

## Abstract

Release of ferulic acid from surface-functionalized hollow nanoporous silica particles (HNSPs) was investigated in deionized water (DI water) and in ethanol. The host material, an HNSP, was synthesized in the presence of polymer and surfactant templates, and the pore as well as the surface were modified with either pentyltriethoxysilane (PTS) or octyltriethoxysilane (OTS) through silane coupling reactions. The inner hollow space occupied a volume of ~45% of the whole HNSP with a 2.54 nm pore channel in the wall. The pore size was estimated to decrease to 1.5 nm and 0.5 nm via the PTS and OTS functionalization, respectively. The encapsulation efficiencies of the HNSP (25 wt%), PTS-functionalized HNSP (PTS-HNSP, 22 wt%) and OTS-functionalized HNSP (OST-HNSP, 25 wt%) toward ferulic acid were similar, while the %release in DI water and ethanol varied following HNSP > PTS-HNSP > OTS-HNSP. Release kinetic analyses with Korsmeyer–Peppas fitting suggested a trade-off relationship between the solvent’s ability to access the HNSP and the affinity of ferulic acid to the surface, allowing us to understand the solvent’s controlled release rate and mechanism.

## 1. Introduction

Over the last few decades, nanotechnology-based drug delivery carriers have been intensively studied in order to enhance drug efficacy [1,2,3] and to minimize side effects [4,5,6]. Various nanomaterials such as liposomes [7], polymer nanoparticles [8], 2-dimensional nanomaterials [9,10] and porous nanomaterials [2] have been under development for these purposes. Indeed, drug delivery by liposomes was found to suppress immune reactions to induce vaccination against COVID-19 [11,12,13]. Among the carriers, porous nanomaterials have attracted great attention due to their characteristic properties such as ordered porosity, large surface areas and modifiable pores.

**Figure 1:**
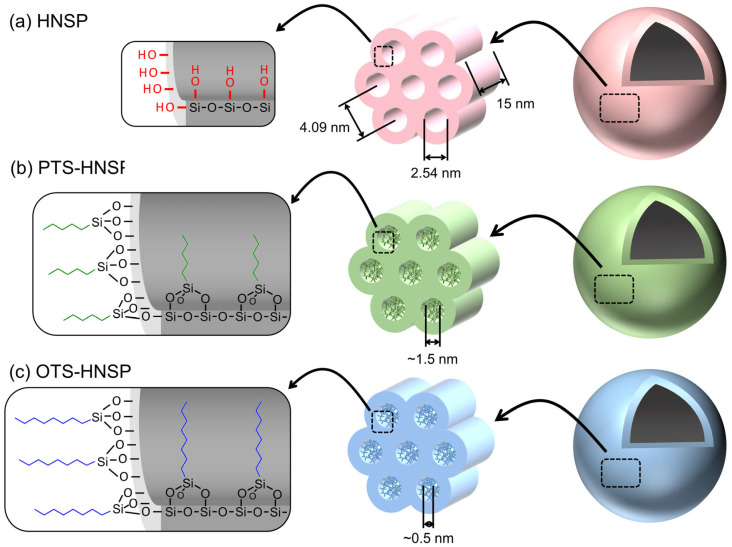
Schematic diagrams of (**a**) HNPS, (**b**) PTS-HNPS and (**c**) OTS-HNSP.

Among the above-mentioned carrier platforms, hollow nanoporous silica particles (HNSPs) are promising materials that can be applied in a field of controlled release thanks to their large inner space and nanopores in their shell [14,15,16,17,18]. It is expected that the hollow inner space of HNSPs can incorporate more drug molecules than conventional porous particles [19,20]. The nanopores in the shell can be modified not only to have a controlled size [14,21] and surface energy [14,20,22,23,24,25], but also to introduce pH [26,27,28,29], chemical [26,30], photo- [26,31] and thermal responses [29,30]. Thus, drug release through modified pores can be either accelerated or sustained. As the chemical nature of pore vicinity is not different from that of conventional nanoporous metal oxides and silicas, various up-to-date surface modification methods that have been reported for metal oxides [32,33,34,35,36,37,38] and silicas [39,40,41] can be applied to modify the nanopores of HNSPs [42,43,44]. The introduction of various lengths of alkyl chains to the pore entrances changed not only the surface energy of the HNSPs, but also their pore size; meanwhile, of the other properties of the HNSPs, i.e., the inner hollow space, were preserved (Figure 1). In terms of sustained release, the correlation between the pore and the release media means that they are both important factors. In our previous studies, organic moieties were grafted on a nanoporous zirconia membrane through a silane coupling reaction to investigate the relationship between the surface energy of the pore entrance and the type of solvent in terms of diffusion across the membrane [32,33].

Herein, we have prepared an HNSP utilizing a sol–gel synthesis method that includes polyvinylpyrrolidone-10 (PVP-10) with dodecylamine (DDA) as a micellar sphere template and tetraethylorthosilicate (TEOS) as a source of the ordered porous silica shell. Furthermore, the pore window of the prepared HNSP was organically modified with either pentyltriethoxysilane (PTS) or octyltriethoxysilane (OTS) through silane coupling reactions (designated as PTS-HNSP and OTS-HNSP, respectively, as shown in Figure 1). To evaluate the loading and the release properties of the HNSP and the organically grafted ones, ferulic acid (FA), which is a model drug, was loaded into the HNSPs and the release of FA was observed under solvents with different surface energies: deionized (DI) water and ethanol. FA was selected as the model drug not only due to its small and compact dimensions, but also due to its wide applicability in biological fields. In fact, FA is known to play several roles, acting as an antioxidant [45]; an anticancer agent [46]; antiviral [47], antibacterial and anti-inflammatory agents [48]; a free-radical scavenger [49,50]; and hepatoprotective [51] and neuroprotective agents [52]. Additionally, it can be used to reduce lipid peroxidation [53] and signal transduction in biological systems [54,55]. The release of FA from carriers has been analyzed with several models, such as zero- and first-order, second-degree polynomial, Elovich, Higuchi, Korsmeyer–Peppas, and Hixson–Crowell models [56,57,58,59]. In this study, the release of FA from the HNSPs was analyzed with four equations, including Fickian (zero order), Korsmeyer–Peppas, Elovich and Hixson–Crowell equations.

## 2. Results and Discussion

**Figure 2:**
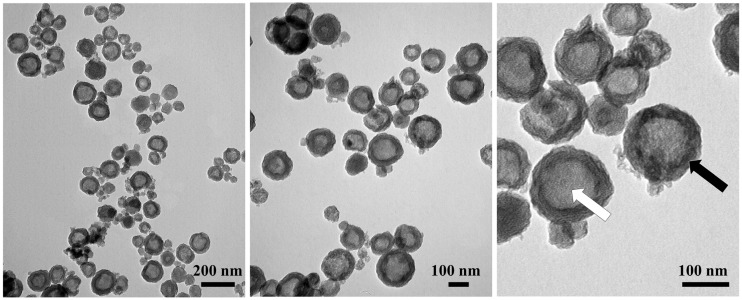
Transmission electron microscope (TEM) images of HNSP particles.

The size and hollow nature of the HNSP were first evaluated with transmission electron microscopy (TEM). As shown in Figure 2, the HNSP has an average particle size of 130 nm and a sphere-like shape with a hollow inner space (white arrow) and silica layers (black arrow). The color contrast between the hollow inner space and the edge of the particle indicates that the HNSP had an inner space diameter of ~100 nm and an outer shell with a 15 nm thickness. The thickness of the outer shell, which interacts with loaded guests mainly, seemed to be relatively irrespective of the particle size. According to the theoretical calculation, the inner space took up 45% of the volume of the whole HNSP particle, suggesting that the inner space of the HNSP was sufficiently large enough to accommodate target molecules (e.g., the size of FA: 1.01 nm × 0.63 nm) [60].

**Figure 3:**
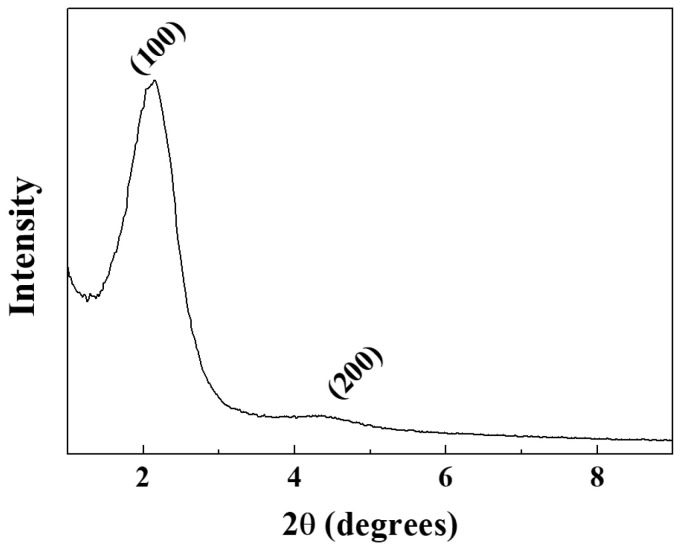
Small-angle X-ray scattering (SAXS) pattern of HNSP.

The small-angle X-ray scattering (SAXS) pattern of the HNSP is shown in Figure 3. Diffraction peaks at 2.16° and 4.28° were attributed to the (100) and (200) plane, indicating the mesoporosity of the HNSP. According to the (100) peak, the interplanar spacing which corresponds to the interpore distance was estimated to be 4.09 nm. The interplanar space suggests that the mesopores of the HNSP were well arranged with 4.09 nm intervals, as shown in Figure 1a.

**Figure 4:**
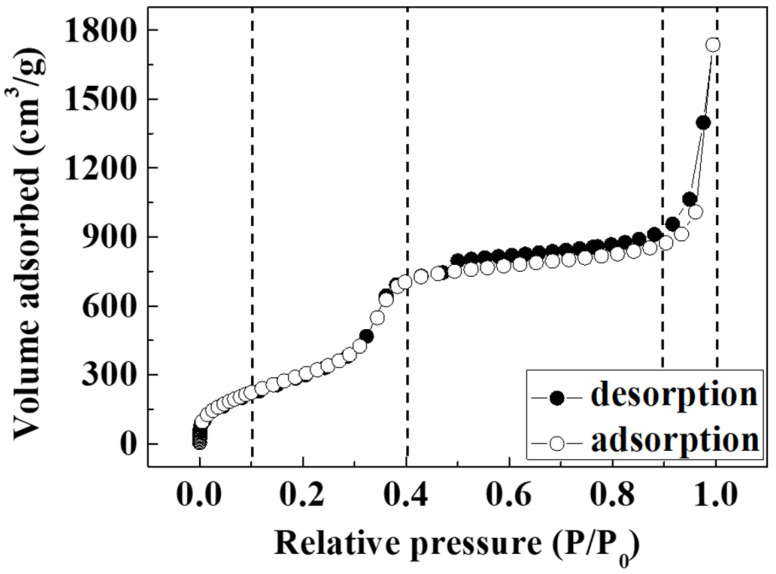
N_2_ adsorption–desorption isotherm of HNSP.

**Figure 5:**
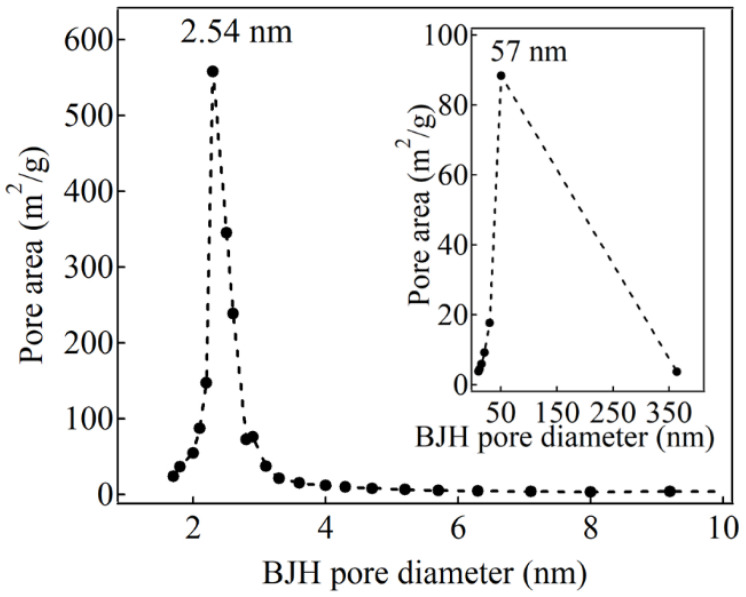
BJH pore size distribution of HNSP.

The N_2_ adsorption–desorption isotherm of the HNSP was a type IV isotherm with a sharp capillary condensation step at a relative pressure (P/P_0_) of 0.3~0.4 (Figure 4) due to its well-developed mesopores. The isotherm observed at the P/P_0_ range from 0 to 0.1 was attributed to single-layer adsorption of N_2_ on the surface of the HNSP, and that between 0.9 and 1.0 was attributed to adsorption into the macropores of the hollow inner space of the HNSP. A similar adsorbed volume for the adsorption–desorption isotherm in the plateau region (0.4 < P/P_0_ < 0.9) suggests that the mesopores of the HNSP are uniformly sized. As summarized in Table 1, the Brunauer–Emmett–Teller (BET) specific surface area of the HNSP was estimated to be 1328 m^2^/g, where the high specific surface area indicated the effective removal of the templates. The pore diameter and pore volume (*V_p_*) of the HNSP were calculated to be 2.54 nm and 1.983 cm^3^/g by the Barrett–Joyner–Halenda (BJH) method, respectively. These results show the higher surface area than that of MCM-41s [45,61,62], which had a surface area around 1000 m^2^/g and pore volume around 1.0 cm^3^/g while having a similar pore diameter (3.16–4.80 nm). Furthermore, the size distribution for mesopores obtained from the BJH method was very narrow (Figure 5), suggesting the homogeneous development of the mesopores at the surface of the HNSP. As the size of primary mesopores (*W_d_*) is 2.54 nm, the thickness between pores (*b_d_*) in the shell was estimated to be 2.30 nm by Equation (1) [63]; these data are displayed in Table 1.

(1) $$b_{d}=2\left(3^{-\frac{1}{2}}\right)d-W_{d}/1.05$$

where *d* is the interplanar space from the (100) peak as estimated from the XRD pattern.

As shown in the inset of Figure 5, the BJH pore size distribution showed a peak at around ~57 nm. Although it is not usual to interpret large pores with the BJH method, we could at least estimate that the peak was attributed to the large hollow space in the HNSP, which was also verified with the TEM measurement (Figure 2). It is important to mention that the hollow inner space of the HNSP made it possible for it to have a higher BET surface area and larger pore volume than MCM-41 with comparable mesopores, and a high loading capacity should be expected by utilizing the suitable leading methods. We are fairly sure that the characteristic porous structure of the HNSP—the large specific surface area and distinctive pore structure with the large hollow space and ordered mesopores in the wall—would have advantages in the loading and release of bioactive molecules compared with the other porous materials with simple pore types.

**Figure 6:**
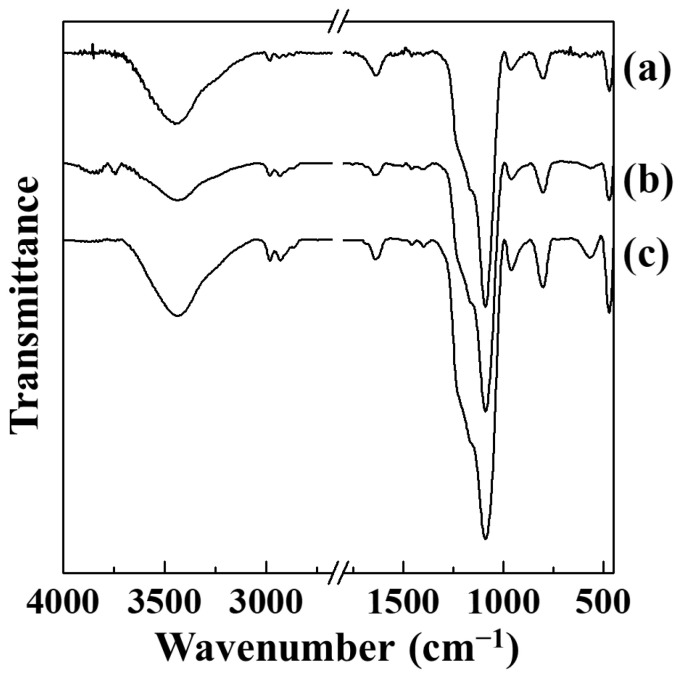
IR spectra of (**a**) HNSP, (**b**) PTS-HNSP and (**c**) OTS-HNSP.

The grafted PTS and OTS moieties of PTS-HNSP and OTS-HNSP were investigated with Fourier transform infrared (FT-IR) spectroscopy (Figure 6). All three samples—the HNSP, PTS-HNSP and OTS-HNSP—showed broad peaks of Si–O–Si asymmetric stretching vibration at 1100 cm^−1^ and Si–O–Si bending vibration at 800 cm^−1^. Other characteristic vibrations, including Si–O rocking vibration and Si-OH vibration, were observed at 460 and 960 cm^−1^. The –CH_2_– stretching vibrations between 1200 and 1500 cm^−1^ and between 2800 and 3000 cm^−1^ were negligibly small in the HNSP, while they were clearly observed in PTS-HNSP and OTS-HNSP at 2980 and 2920 cm^−1^ and were attributed to the alkyl chain moieties in PTS and OTS, suggesting the removal of the templates and the grafting of the HNSP by PTS and OTS. The IR peak patterns of the PTS-HNSP and OTS-HNSP were comparable with the results in which silane moieties were attached to mesoporous silicas such as MCM-41 [64,65,66,67] and SBA-15 [68,69,70]. Therefore, the grafting reactions with PTS and OTS were thought to be successful in this study, and that the attached molecules would modify the pore structure as suggested in Figure 1.

The FA contents in the HNSP, PTS-HNSP and OTS-HNSP were evaluated using the UV–vis absorption spectra of FA in the supernatant. The loading capacities (g-FA/g-FA-loaded HNSPs) of the HNSP, PTS-HNSP and OTS-HNSP were 25 wt%, 22 wt% and 25 wt%, respectively. The FA contents in the HNSP, PTS-HNSP and OTS-HNSP were fairly comparable regardless of the surface modification, suggesting that the surface chemistry of silica neither altered the pore structure nor the encapsulation of FA significantly.

**Figure 7:**
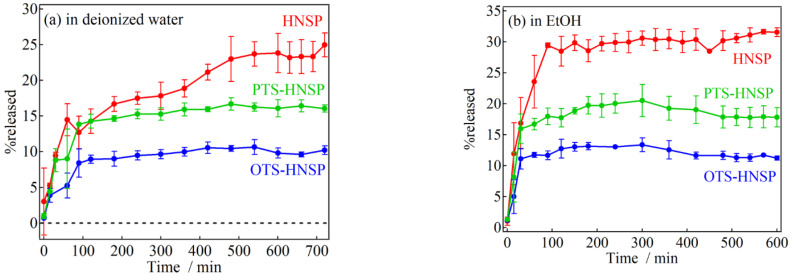
Release profiles of FA from HNSP (red line), PTS-HNSP (green line) and OTS-HNSP (blue line) in (**a**) DI water and (**b**) EtOH at room temperature.

**Figure 8:**
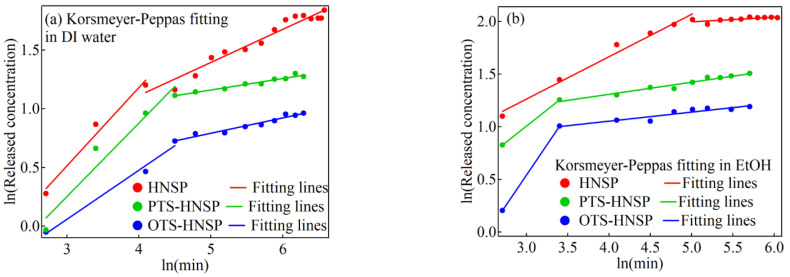
Korsmeyer–Peppas fittings of the release of FA from HNSP (red line), PTS-HNSP (green line) and OTS-HNSP (blue line) in (**a**) DI water and (**b**) EtOH.

In order to investigate the effects of the surface modification on the release profiles of FA from the HNSPs, two different solvents (either DI water or EtOH) with different surface tensions (γLV; water = 72.8 mN/m and EtOH = 22.1 mN/m) and solubilities of FA (water: 6.25 × 10^−5^ and EtOH: 2.54 × 10^−2^ in mole fraction at 303.2 K) [71] were used. In these solvents, the HNSPs without FA loading were precipitated in 4 h after short ultrasonication, although the precipitation ratio was slightly different for the surface functionalization and the solvent. As shown in Figure 7a, FA in DI water showed %release (=released FA (mg)/encapsulated FA (mg) × 100) of ~25%, ~16% and ~10% after 10 h for the HNSP, PTS-HNSP and OTS-HNSP, respectively. The %release in EtOH was higher than that in DI water (HNSP: ~32%, PTS-HNSP: ~21% and OTS-HNSP: ~13%) due to the higher solubility of FA in EtOH (Figure 7b). In both cases, the %release showed the order of HNSP > PTS-HNSP > OTS-HNSP, which corresponded to the diameter of the pores. The release kinetics were not fully understood by concentration-gradient-driven Fickian transport (Equation (2)), as shown in Figures S1 and S2 in the Supplementary Materials, and thus the modified release was hypothesized using the distinct pore structure of the HNSPs (Figure 8 and Figures S3–S6 in the Supplementary Materials). The releases were further analyzed by fitting with the Korsmeyer–Peppas (Equation (3)) [72], Elovich (Equation (4)) [58] and Hixson–Crowell (Equation (5)) [57,73] equations.

(2) $$Q_{t}=k_{H}t^{\frac{1}{2}}$$

(3) $$\ln\left(\frac{Q_{t}}{Q_{inf}}\right)=\ln\left(k_{KP}\right)+n\;ln(t)$$

(4) $$Q_{t}=\left(\frac{1}{b}\right)\ln\left(ab\right)+\left(\frac{1}{b}\right)ln(t)$$

(5) $$M_{0}^{1/3}-M_{t}^{1/3}=k_{HC}t$$

where *Q_t_* and *Q_inf_* are the released amount (mg/L) of FA at time *t* and ∞ (min); *k_H_*, *k_KP_* and *k_HC_* are the release rate (mg L^−1^ min^−1/2^), a proportionality constant (min^−*n*^) and a constant related to the morphology of the carrier (mg^1/3^ min^−1^); *n* is the release index; *a* is the initial adsorption rate (mg/(L min)); *b* is the Elovich constant (L/mg); and *M_0_* and *M_t_* are the amount of FA in HNSPs (mg) at the initial condition and at time *t*.

Although the χ^2^ values obtained by the Hixson–Crowell fittings were the smallest due to the small y-axis values, the Korsmeyer–Peppas model seemed to be the best among the applied models to explain the release kinetics (Figure 8 and Figures S1–S8 and Tables S1–S6 in the Supplementary Materials). The fitting parameters are summarized in Table 2. The release of FA from the HNSPs clearly included two steps, with larger *n* values in the first step and smaller *n* values in the second step. The *n* value less than 0.43 indicates a drug release mechanism of Fickian diffusion; the *n* value between 0.43 and 0.85 suggests non-Fickian transport, and *n* = 0.85 leads to a zero-order release induced by swelling or dissolution of the carrier in the sphere morphology [74]. It has been shown that the release of chemicals from some mesoporous silicas involves two steps [2,14,16,75,76,77,78]. The turnover in the release processes within the HNSPs was clearer than that in previous reports. In DI water, the *n* values decreased, following HNSP > PTS-HNSP > OTS-HNSP, while those in EtOH had the opposite trend (OTS-HNSP > PTS-HNSP > HNSP). In addition to the solubility of FA and the surface tension of the solvent, three factors were thought to affect the release kinetics: (i) the pore size of carriers (a larger pore accelerates the release [79]), (ii) the wettability of the carrier surface due to the solvents (a higher wettability accelerates the release by effective swelling [74]), and (iii) the affinity of FA toward the carrier surface (a lower affinity accelerates the release). The DI water’s access to the carriers is thought to follow the trend HNSP > PTS-HNSP > OTS-HNSP; this is due to the pore size as well as the hydrophilic/-phobic nature of these three carriers, resulting in the most accelerated release of FA from the HNSP. On the other hand, the affinity of FA to the silanol at the HNSP surface is higher than the affinity of FA to PTS and OTS, negatively affecting the release of FA from the HNSP. This trade-off relationship between the accessibility of DI water and the adsorption affinity of FA led to the trend in the first steps with the similar *n* values in the HNSP and PTS-HNSP. The smallest pore size and lowest water accessibility for the OTS-HNSP gave the smallest *n* value. The higher solubility of FA in EtOH than that in DI water increased the *n* values in EtOH. The ability of EtOH to access the pores of the HNSP seems to be slightly higher than the others owing to the hydrophilicity and the large pore size, while the acceleration of the release due to the lower affinity of FA toward the OTS-HNSP than that toward the HNSP seemed to have a major effect on the large *n* value in the OTS-HNSP system. The quick permeation of the solvents was thought to cause the burst release in the first steps with non-Fickian diffusion, which is a kind of bottleneck effect. In the second step, all of the *n* values were less than 0.3, which indicates that the second step followed Fickian diffusion, although the same trends as in the first steps were observed. Both in the DI water and EtOH suspensions, the *k_KP_* values, which are thought to be related to the release rate, followed the trend of HNSP > PTS-HNSP > OTS-HNSP in the first steps, suggesting that the larger pore size contributed to a better release as previously reported [76,79,80].

## 3. Materials and Methods

### 3.1. Materials

Reagent-grade 98% Si(OC_2_H_5_)_4_ (tetraethylorthosilicate, TEOS), (C_6_H_9_NO)_n_ (polyvinylpyrrolidone-10, MW ≈ 10,000, PVP-10), 98% CH_3_(CH_2_)_10_CH_2_NH_2_ (dodecylamine, DDA), 99.8% C_7_H_8_ (toluene) and 99% C_10_H_10_O_4_ (ferulic acid, FA) were purchased from Sigma-Aldrich Co., LLC (St. Louis, MO, USA); 99.9% CH_3_CH_2_OH (ethanol, EtOH) was obtained from Burdick and Jackson (Charlotte, NC, USA); >95.0% C_11_H_26_O_3_Si (pentyltriethoxysilane, PTS) and C_14_H_32_O_3_Si (octyltriethoxysilane, OTS) were purchased from Tokyo Chemical Industry Co., Ltd. (Tokyo, Japan). All of the reagents were used without further purification.

### 3.2. Synthesis of Hollow Nanoporous Silica Particles (HNSPs)

The polymer template, PVP-10 (0.1 g), was dissolved in 20 mL of 20% EtOH aqueous solution and was vigorously stirred for 1 h. Subsequently, 1.17 g of DDA was added to the PVP-10 solution at room temperature and stirred for 30 min. Then, 5 mL of TEOS was added to the solution and stirred for another 24 h at room temperature to cause the sol–gel reaction for the silica. The obtained precipitate was centrifuged and dried in a vacuum at 40 °C to obtain white silica particles. The silica particles (1.0 g) were dispersed in 100 mL of EtOH and refluxed at 80 °C for 24 h under vigorous stirring to remove the PVP-10 sphere and the DDA template to obtain the HNSP. The product was washed with EtOH several times and dried in a vacuum at 40 °C.

In order to control the pore size and surface energy of the HNSP, the surface of the HNSP was grafted with PTS and OTS, respectively [32,33]. The HNSP (1.0 g) was placed in an empty flask and the flask was heated at 70 °C under a vacuum condition to remove the water adsorbed on the surface and inside of the HNSP. After filling the flask with N_2_ gas, 100 mL of dried toluene was added to the flask through a rubber septum. Then, either PTS or OTS (5 mL) was injected into the reaction media and the reaction mixture was refluxed at 120 °C for 12 h. The obtained powder was washed with toluene three times to remove unreacted PTS and OTS and dried in a vacuum at 40 °C. The PTS- and OTS-grafted HNSPs were designated as the PTS-HNSP and OTS-HNSP, respectively.

### 3.3. Ferulic Acid Loading and Release

The organically grafted HNSPs and the pristine HNSP were dispersed into FA solutions (20 g/L in EtOH) and stirred for 5 h (90 rpm) at room temperature. The resulting powder was collected by centrifugation. The concentration of FA in the supernatant was estimated by UV–vis absorption spectroscopy (UV–vis spectrometer (SHIMADZU, Kyoto, Japan, UV-1800) at 320 nm) to calculate the amount to be loaded into the HNSPs. The final products were dried at 40 °C in a vacuum for 12 h. The time-dependent release of FA was performed by adding 50 mg of the FA-loaded HNSP, PTS-HNSP and OTS-HNSP to either 500 mL of EtOH or DI water. In order to exclude the effect of particle aggregation in the FA release, all of the HNSPs were thoroughly dispersed in each medium (water or EtOH) through stirring and short ultrasonication prior to the release test. An aliquot (1.75 mL) was collected at designated time points. The dispersion of the aliquot was filtered with a syringe filter (0.45 μm pore, polytetrafluoroethylene) and the concentration of FA was quantified by UV–vis absorption spectroscopy. The %release (= released FA (mg)/encapsulated FA (mg) × 100) was calculated to analyze the release kinetics.

### 3.4. Characterization

The removal of templates from the HNSP and organic functionalization of the HNSP were confirmed by Fourier transform infrared spectroscopy (FT-IR; PerkinElmer, Waltham, MA, USA, spectrum one B.v5.0) by KBr methods. The N_2_ adsorption isotherm was measured with the ASAP2020 (Micromeritics Instrument Corp., Norcross, GA, USA) to estimate pore size and the specific surface area. The interpore distance of the HNSP was estimated from small-angle X-ray scattering (SAXS) patterns that were taken with the Bruker GADDS, and from wide-angle X-ray scattering (WAXS) patterns that were taken with the Bruker D2 Phaser. The particle size and inner hollow space of the HNSP were investigated with transmission electron microscopy (TEM) via a JEOL1010 (JEOL, Tokyo, Japan).

## 4. Conclusions

We successfully demonstrated the controlled release of FA by utilizing the pore properties of carriers (pore sizes of 2.5 nm, 1.5 nm and 0.5 nm and surface functionalization with silanol, pentylsilane and octylsilane, respectively) and the release media (DI water and EtOH). First of all, the pore size exhibited a strong influence on the release rate, showing a higher release in the larger pores. Surface functionalization and the solvent used also controlled the %release at equilibrium. FA was released up to 31% in ethanol in 700 min, which was higher than in deionized water owing to the better solubility of FA in EtOH than in DI water. The release profiles were analyzed by four models, including the Fickian, Korsmeyer–Peppas, Elovich and Hixson–Crowell models. The two steps of the release were clearly observed in Korsmeyer–Peppas fitting and were attributed to the bottle-neck effect, causing: (i) a direct non-Fickian release from the surface and the mesopores by solvent permeation, and (ii) an indirect release from the hollow inner space by Fickian diffusion. The major controlling factors of the release mechanism in DI water were thought to be pore size and the ability of the DI water to access the pore, and those in EtOH were pore size and the stabilization of FA. Therefore, the release kinetics were controlled by pore size and the interaction among three components: the solvent, the surface function of the carriers and the cargo molecule (FA). As the next step to utilize HNSPs as practical drug delivery carriers, further studies on colloidal stability and biocompatibility in various biological media will be performed.

## Figures and Tables

**Table 1:** Structural data of HNSP.

	*d*_100_ (nm)	*b_d_* (nm)	BET Surface Area (m^2^/g)	BJH Pore Size, *W_d_* (nm)	*V_p_* (cm^3^/g)
HNSP	4.09	0.46	1328	2.54	1.983

**Table 2:** Fitting parameter of Korsmeyer–Peppas equation of HNSP, PTS-HNSP and OTS-HNSP in DI water and EtOH.

Solvent	Carrier	*k_KP_* (min^−*n*^)	*n*	*χ* ^2^
DI water	HNSP	0.227	0.666	0.0110
		0.984	0.282	0.0360
	PTS-HNSP	0.197	0.626	0.0425
		1.95	0.098	1.25 × 10^−3^
	OTS-HNSP	0.0303	0.418	0.00426
		1.15	0.130	2.15 × 10^−3^
EtOH	HNSP	0.943	0.402	0.0119
		5.93	0.0435	1.70 × 10^−3^
	PTS-HNSP	0.426	0.620	1.97 × 10^−30^
		2.33	0.166	2.56 × 10^−3^
	OTS-HNSP	0.0536	1.16	1.97 × 10^−30^
		2.03	0.0858	3.57 × 10^−3^

## Data Availability

The data presented in this study are available upon request from the corresponding author. The data are not publicly available due to privacy or ethical restrictions.

## Supplementary Materials

The following supporting information can be downloaded at: https://www.mdpi.com/article/10.3390/ijms241310560/s1.
