# Supplementary material for: Time-Dependent Controlled Release of Ferulic Acid from Surface-Modified Hollow Nanoporous Silica Particles
Source: Int J Mol Sci. 2023 Jun 23;24(13):10560. doi: 10.3390/ijms241310560 (PMC10342040; doi:10.3390/ijms241310560)
Supplement: Supplementary file 1 [file ijms-24-10560-s001.zip › ijms-2434479-supplementary.pdf]

Supporting Information for:

Time dependent controlled release of ferulic acid from surface modified hollow nanoporous silica particles

Tetsuo Yamaguchi,[1] Taeho Kim[1] Jin-Kuen Park[2]*, and Jae-Min Oh[1*]

[1]Department of Energy and Materials Engineering, Dongguk University-Seoul, Seoul 04620, Korea
2Department of Chemistry, Hankuk University of foreign studies, Gyeonggi-do, Korea

E-mail: J.-M. Oh *jaemin.oh@dongguk.edu*
J.-K. Park *jinkuenpark@gmail.com*

2. Adsorption isotherms

Fickian model

In deionized water

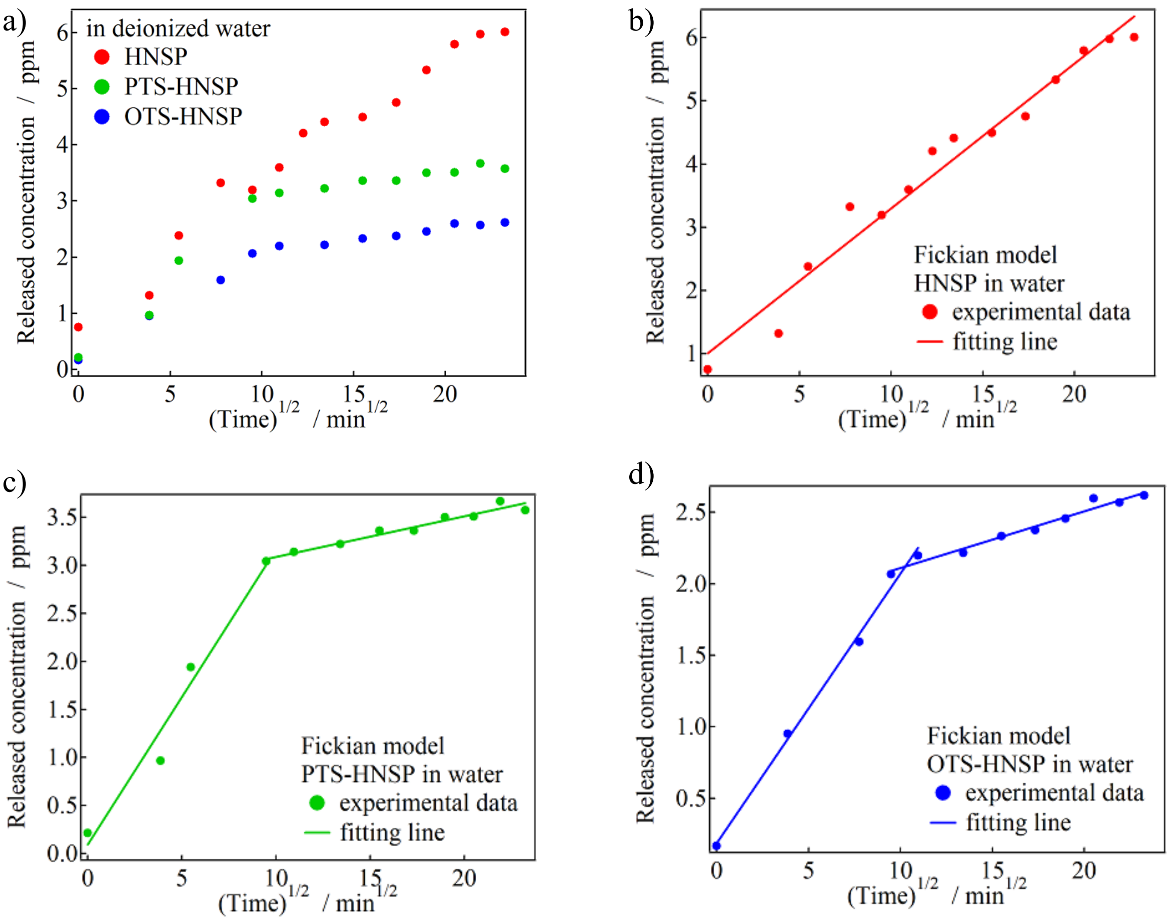

Figure S1 a) Fickian plots of time dependent FA release in deionized water and (b-d) fitting lines with Fickian equation mentioned in equation 2 in the main manuscript of b) HNSP, c) PTS-HNSP and d) OTS-HNSP.

Table S1. Fitting parameters for Fickian equation of HNSP, PTS-HSNP and OTS-HNSP in DI water.

Carrier	k_H	χ^2
HNSP	0.222	1.04
PTS-HNSP	0.358	0.0998
	0.0423	0.0164
OTS-HNSP	0.183	0.0144
	0.0398	0.00996

In EtOH

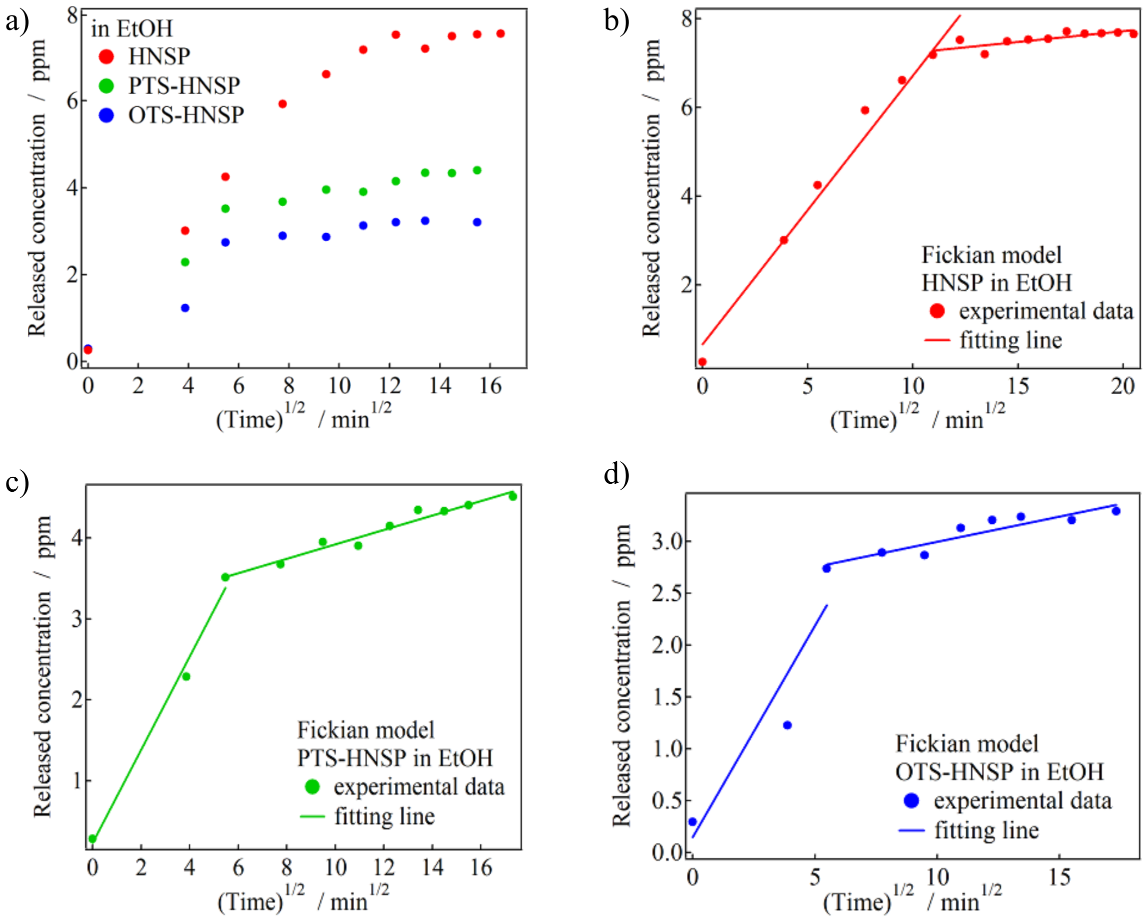

Figure S2 a) Fickian plots of time dependent FA release in EtOH and (b-d) fitting lines with Fickian equation mentioned in equation 2 in the main manuscript of b) HNSP, c) PTS-HNSP and d) OTS-HNSP.

Table S2. Fitting parameters for Fickian equation of HNSP, PTS-HSNP and OTS-HNSP in EtOH.

Carrier	k_H	χ^2
HNSP	0.605	0.929
	0.0487	0.109
PTS-HNSP	0.576	0.0500
	0.0892	0.0368
OTS-HNSP	0.408	0.397
	0.0487	0.00427

Korsmeyer-Peppas model

In deionized water

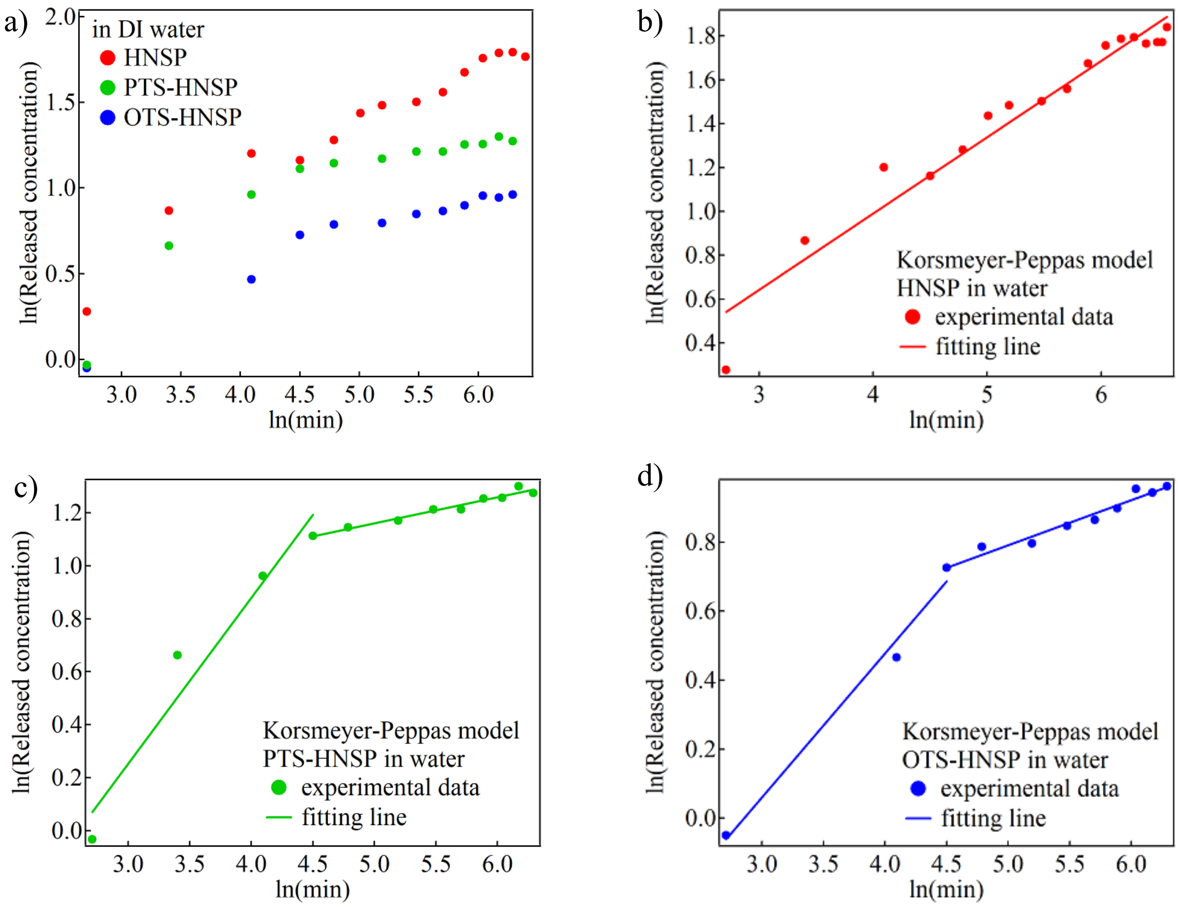

Figure S3 a) Korsmeyer-Peppas plots of time dependent FA release in deionized water and (b-d) fitting lines with Korsmeyer-Peppas equation mentioned in equation 3 in the main manuscript of b) HNSP, c) PTS-HNSP and d) OTS-HNSP.

In EtOH

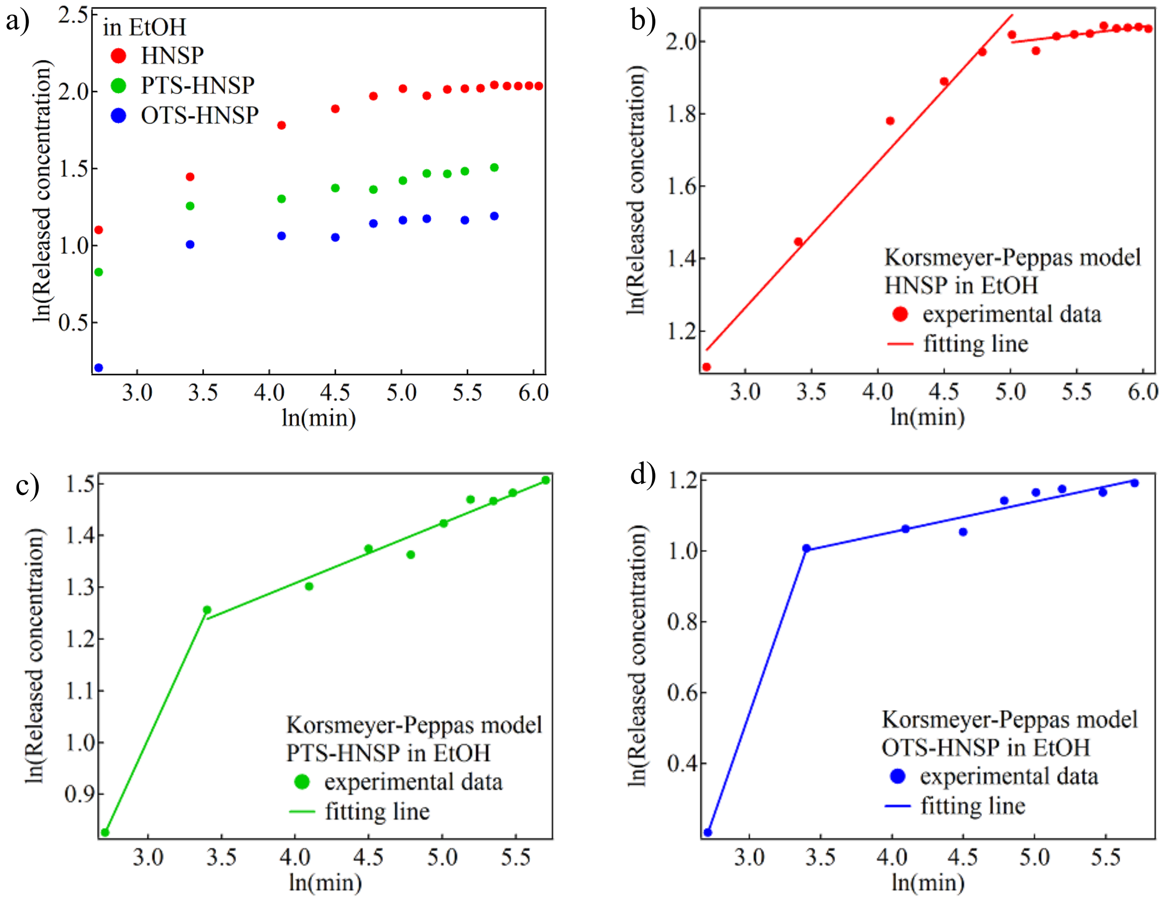

Figure S4 a) Korsmeyer-Peppas plots of time dependent FA release in deionized water and (b-d) fitting lines with Korsmeyer-Peppas equation mentioned in equation 3 in the main manuscript of b) HNSP, c) PTS-HNSP and d) OTS-HNSP.

Elovich model

In deionized water

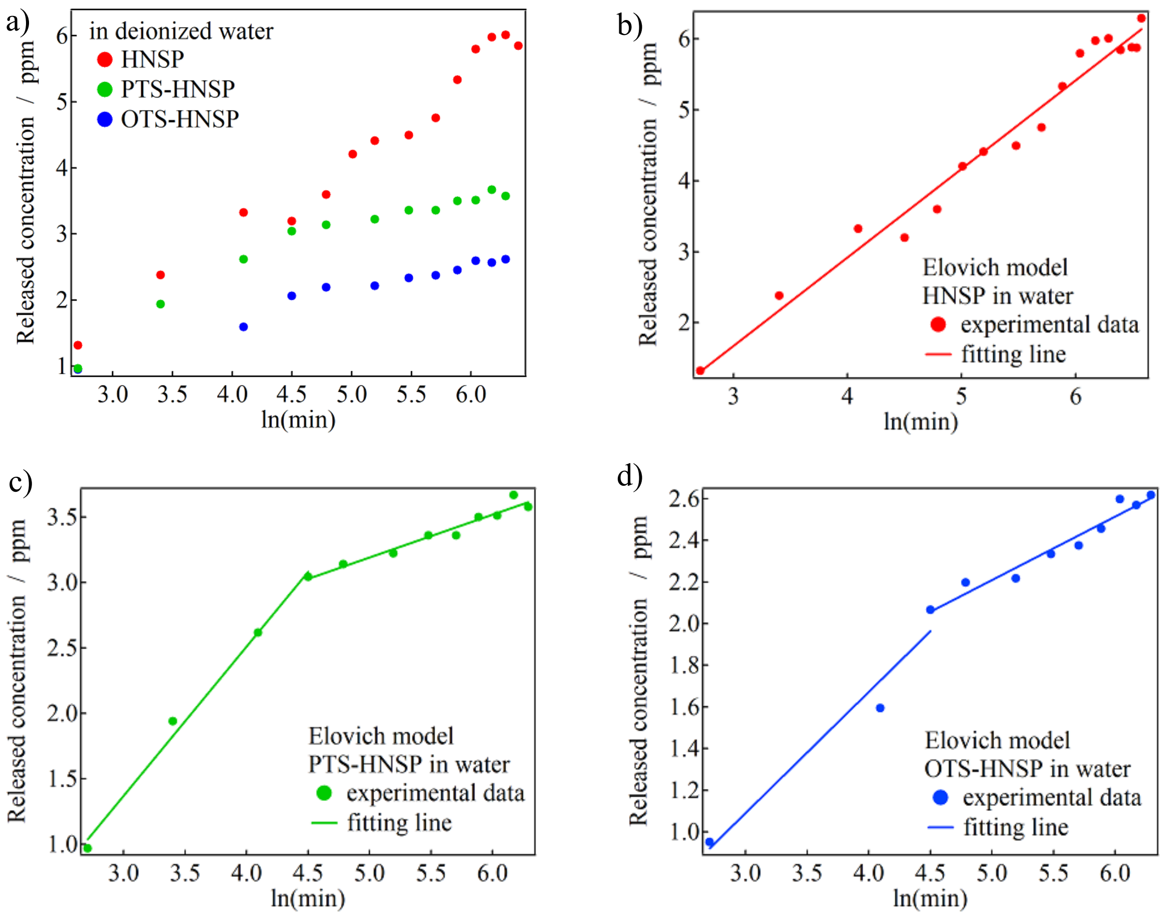

Figure S5 a) Elovich plots of time dependent FA release in deionized water and (b-d) fitting lines with Elovich equation mentioned in equation 4 in the main manuscript of b) HNSP, c) PTS-HNSP and d) OTS-HNSP.

Table S3 Fitting parameters for Elovich equation of HNSP, PTS-HSNP and OTS-HNSP in DI water.

Carrier	*a*	*b*	χ^2
HNSP	0.238	0.802	0.873
PTS-HNSP	0.189	0.875	0.0189
	38.4	3.04	0.0164
OTS-HNSP	0.188	1.72	0.110
	2.86	3.27	0.014

In EtOH

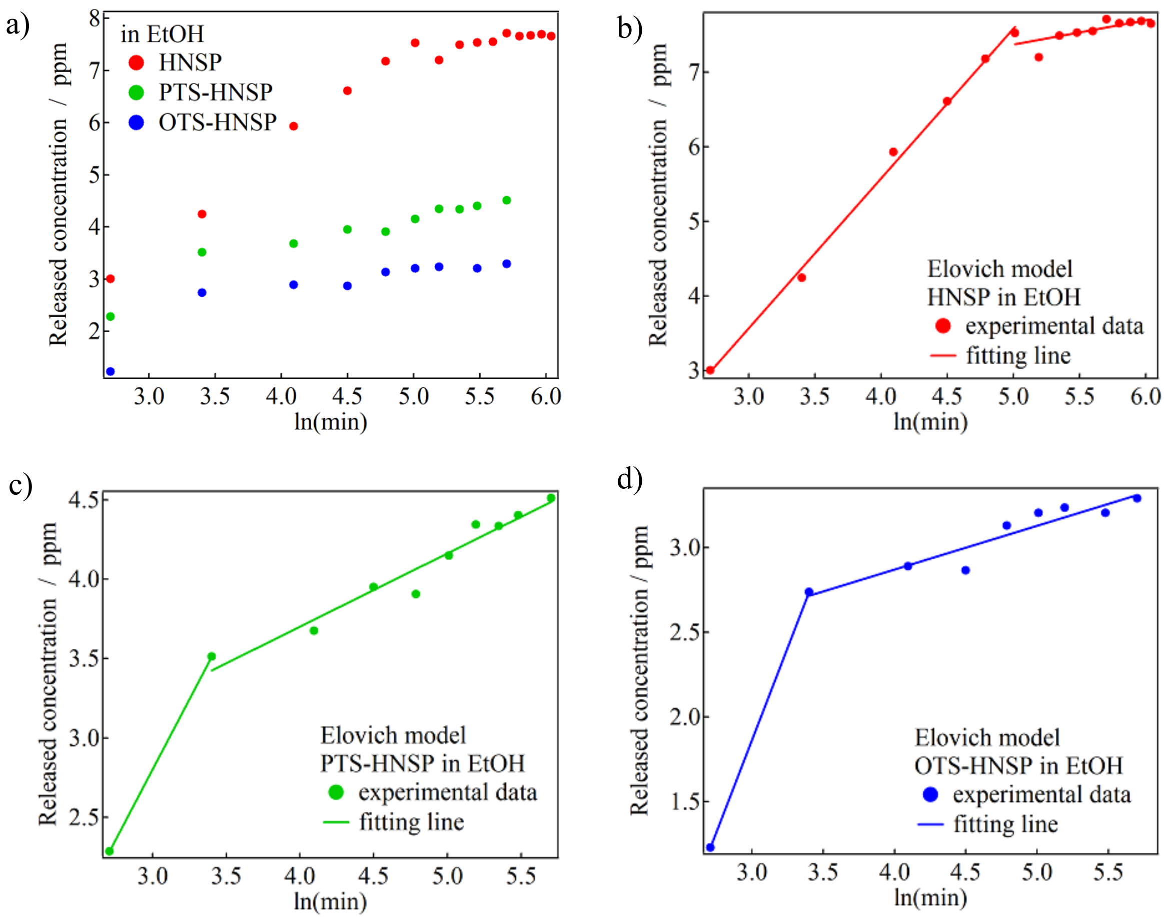

Figure S6 a) Elovich plots of time dependent FA release in EtOH and (b-d) fitting lines with Elovich equation mentioned in equation 4 in the main manuscript of b) HNSP, c) PTS-HNSP and d) OTS-HNSP.

Table S4 Fitting parameters for Elovich equation of HNSP, PTS-HSNP and OTS-HNSP in EtOH.

Carrier	a	b	χ^2
HNSP	0.592	0.497	0.0521
	1.38×10^{7}	3.06	0.0935
PTS-HNSP	0.429	0.565	1.02×10^{-29}
	25.9	2.17	0.0481
OTS-HNSP	0.255	0.459	1.02×10^{-30}
	317	3.87	0.0328

Hixson-Crowell model

In deionized water

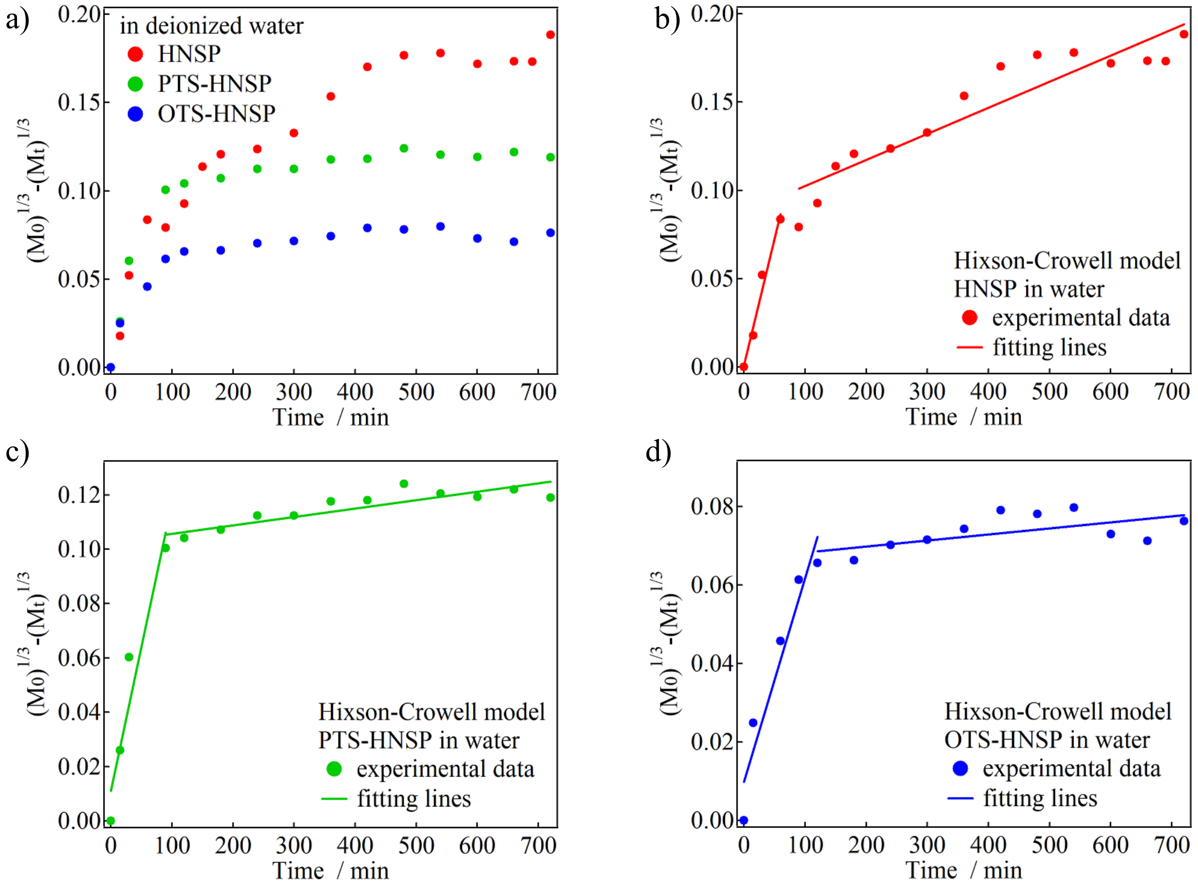

Figure S7 a) Hixson-Crowell plots of time dependent FA release in deionized water and (b-d) fitting lines with Hixson-Crowell equation mentioned in equation 5 in the main manuscript of b) HNSP, c) PTS-HNSP and d) OTS-HNSP.

Table S5 Fitting parameters for Hixson-Crowell equation of HNSP, PTS-HSNP and OTS-HNSP in deionized water.

Carrier	k_{HC}	χ^2
HNSP	0.00143	0.00101
	1.48×10^{-4}	2.16×10^{-4}
PTS-HNSP	0.00110	4.61×10^{-4}
	3.09×10^{-5}	1.42×10^{-4}
OTS-HNSP	5.21×10^{-4}	2.37×10^{-4}
	1.53×10^{-5}	1.39×10^{-4}

In EtOH

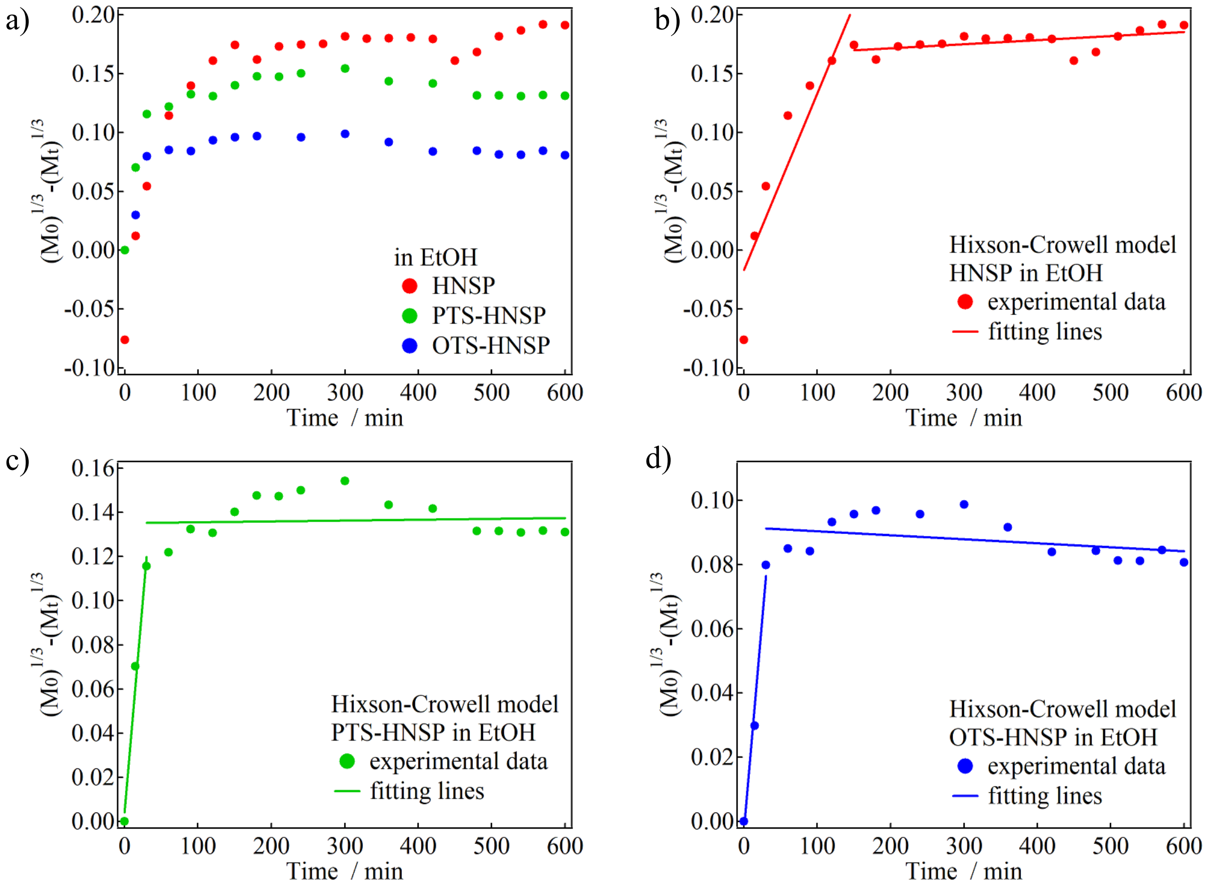

Figure S8 a) Hixson-Crowell plots of time dependent FA release in EtOH and (b-d) fitting lines with Hixson-Crowell equation mentioned in equation 5 in the main manuscript of b) HNSP, c) PTS-HNSP and d) OTS-HNSP in EtOH.

Table S6 Fitting parameters for Hixson-Crowell equation of HNSP, PTS-HSNP and OTS-HNSP in EtOH.

Carrier	k_{HC}	χ^2
HNSP	0.00150	0.00752
	3.4×10^{-5}	8.16×10^{-4}
PTS-HNSP	0.00386	1.02×10^{-4}
	3.86×10^{-6}	0.00165
OTS-HNSP	0.00266	6.74×10^{-5}
	−1.25	5.45×10^{-4}
